# Early systemic inflammatory indices and postoperative SIRS after percutaneous nephrolithotomy under spinal versus general anaesthesia: a prospective cohort study

**DOI:** 10.1186/s12894-026-02368-y

**Published:** 2026-09-19

**Authors:** Sait Fatih Öner, Sevim Şenol Karataş, Oğuz Kağan Bulut, Kadir Yıldırım

**Affiliations:** 1 Department of Anaesthesiology, University of Health Sciences, Elazig Fethi Sekin City Hospital, Elazig, Turkey; 2 Department of Urology, University of Health Sciences, Fethi Sekin City Hospital, Elazig, Turkey

**Keywords:** Percutaneous nephrolithotomy, Spinal anaesthesia, General anaesthesia, Systemic inflammatory response, Neutrophil-to-lymphocyte ratio

## Abstract

**Objective:**

Percutaneous nephrolithotomy (PCNL) may induce an early systemic inflammatory response. This study compared perioperative changes in systemic inflammatory indices between patients undergoing elective PCNL under spinal or general anaesthesia and explored the relationship of these indices with postoperative systemic inflammatory response syndrome (SIRS).

**Materials and methods:**

This single-centre, prospective, non-randomised observational cohort study included consecutive adult patients undergoing elective PCNL between November 2025 and June 2026. Anaesthetic technique was determined as part of routine clinical care, and patients were classified according to the technique received. The primary outcome was the between-group comparison of perioperative changes in the neutrophil-to-lymphocyte ratio (NLR), systemic immune-inflammation index (SII), systemic inflammation response index (SIRI), and aggregate index of systemic inflammation (AISI), measured preoperatively and 6 h postoperatively. Secondary clinical outcomes were postoperative SIRS, fever, and length of hospital stay. Receiver operating characteristic analysis, decision curve analysis, and a parsimonious two-variable logistic regression analysis involving SIRS were performed as exploratory secondary analyses.

**Results:**

A total of 150 patients were included in the final analysis, with 75 patients in each anaesthesia group. Preoperative inflammatory indices were similar between the groups, whereas postoperative NLR, SII, SIRI, and AISI values and their corresponding Δ values were significantly higher in the general anaesthesia group (all *p* < 0.001). Postoperative SIRS occurred in 5 patients (6.7%) in the spinal anaesthesia group and 16 patients (21.3%) in the general anaesthesia group (*p* = 0.017), while postoperative fever occurred in 10.7% and 24.0%, respectively (*p* = 0.031). Length of hospital stay did not differ significantly between groups. Among the evaluated inflammatory indices, ΔAISI showed the highest discriminatory performance for postoperative SIRS (AUC = 0.962, 95% CI: 0.923–0.991). In the exploratory parsimonious multivariable model, general anaesthesia (adjusted OR = 12.02, 95% CI: 1.06–136.68, *p* = 0.045) and ΔAISI (adjusted OR = 1.51 per 100-unit increase, 95% CI: 1.26–1.81, *p* < 0.001) were associated with postoperative SIRS after mutual adjustment. The wide confidence interval for general anaesthesia indicates substantial imprecision in the magnitude of this association.

**Conclusion:**

General anaesthesia was associated with a more pronounced early postoperative inflammatory response and higher observed rates of SIRS and fever than spinal anaesthesia in this prospective observational cohort. ΔAISI showed the highest discriminatory performance for postoperative SIRS among the evaluated inflammatory indices; however, the SIRS-related analyses were exploratory and based on only 21 events. These findings require confirmation in larger independent cohorts before ΔAISI or its internally derived threshold can be considered for clinical risk stratification.

**Supplementary Information:**

The online version contains supplementary material available at https://doi.org/10.1186/s12894-026-02368-y.

## Introduction

Urolithiasis is a common urological disease with a steadily increasing global incidence and is associated with considerable morbidity [1]. Percutaneous nephrolithotomy (PCNL) is currently regarded as the standard surgical treatment for large, complex, and staghorn renal calculi because of its high stone-free rates and acceptable complication profile [2]. Nevertheless, PCNL can trigger a systemic inflammatory response, and postoperative systemic inflammatory response syndrome (SIRS) has been associated with several perioperative factors [3].

The systemic inflammatory response induced by surgical trauma is closely linked to postoperative complications, prolonged hospitalisation, and delayed recovery [4]. Early and reliable assessment of postoperative inflammation is therefore clinically important for risk stratification and postoperative management. In recent years, complete blood count–derived biomarkers, including the neutrophil-to-lymphocyte ratio (NLR), systemic immune-inflammation index (SII), systemic inflammation response index (SIRI), and aggregate index of systemic inflammation (AISI), have emerged as practical and inexpensive indicators of systemic inflammatory activity [5, 6].

The choice of anaesthetic technique may influence the inflammatory and immune responses to surgery. General anaesthesia has been associated with a more pronounced neuroendocrine stress response, whereas regional anaesthesia may attenuate this response through sympathetic blockade [7, 8]. However, the relative effects of spinal and general anaesthesia on the systemic inflammatory response following PCNL remain uncertain, and the available evidence is limited. In addition, the inflammatory response after PCNL is multifactorial and may be influenced not only by surgical trauma but also by procedure-specific factors, including irrigation-related pressure changes, transient bacteraemia, and endotoxaemia [9]. These factors may complicate interpretation of the association between anaesthetic technique and the postoperative inflammatory response.

The present study evaluated the association between anaesthetic technique and the early postoperative systemic inflammatory profile in patients undergoing PCNL for renal calculi using readily available inflammatory biomarkers, including NLR, SII, SIRI, and AISI.

## Materials and methods

### Study design and ethical approval

This single-centre, prospective, non-randomised observational cohort study was conducted at Elazığ Fethi Sekin City Hospital between November 2025 and June 2026. Ethical approval was obtained from the Clinical Research Ethics Committee of Elazığ Fethi Sekin City Hospital (6 November 2025; Decision No. 2025/18–24). The study was conducted in accordance with the principles of the Declaration of Helsinki. Written informed consent was obtained from all participants before enrolment. Reporting followed the Strengthening the Reporting of Observational Studies in Epidemiology (STROBE) statement. As this was a non-interventional observational cohort in which anaesthetic technique was determined as part of routine clinical care rather than assigned by the investigators, the study was not registered as a clinical trial. Patients were enrolled prospectively, and study variables were recorded during the perioperative and postoperative course rather than being assembled retrospectively from an existing electronic health-record database or registry. Eligibility criteria, scheduled perioperative measurements, the postoperative surveillance framework, and the primary outcome were established before patient recruitment. A separate prespecified detailed statistical analysis plan was not available; therefore, the ROC, decision curve, and multivariable analyses involving postoperative SIRS are explicitly presented as exploratory secondary analyses.

### Study population

Consecutive adult patients scheduled for elective percutaneous nephrolithotomy were prospectively assessed for eligibility. Patients were classified into the spinal or general anaesthesia cohort according to the anaesthetic technique actually received as part of routine clinical care. No randomisation or research-related allocation of anaesthetic technique was performed. The choice of anaesthesia was made by the attending anaesthesiologist on the basis of the patient’s clinical status, comorbidities, surgical requirements, perioperative assessment findings, and patient preference. Accordingly, the study was conducted as a non-randomised observational cohort. After application of the predefined eligibility and exclusion criteria, the final analytical cohort comprised 150 patients, including 75 patients who had received spinal anaesthesia and 75 who had received general anaesthesia; these group sizes reflect the composition of the final eligible cohort and did not result from research-related allocation.

The inclusion criteria were age ≥ 18 years, elective PCNL for renal or upper ureteral calculi, and complete laboratory data available at both the preoperative time point and 6 h postoperatively.

The exclusion criteria were missing laboratory data; percutaneous nephrostomy alone; major surgery or combined endourological procedures performed during the same session; active urinary tract infection; postoperative SIRS that, after clinical adjudication, was attributed to a clearly identifiable infectious focus outside the urinary tract or to a non-infectious alternative cause such as atelectasis, intra-abdominal haematoma, or perirenal haematoma; malignancy; chronic inflammatory or haematological disease; immunodeficiency; immunosuppressive treatment; indwelling urinary catheter; major congenital renal anomalies; massive transfusion likely to affect perioperative laboratory values; protocol deviations; and loss to follow-up.

Sample size was estimated using G*Power software (v3.1.9.7) on the basis of the study’s primary outcome, namely the between-group comparison of perioperative inflammatory change. Because no previous prospective PCNL study had directly compared postoperative complete blood count–derived inflammatory indices between spinal and general anaesthesia, a moderate-to-large standardised effect size (Cohen’s d = 0.70) was assumed. With a two-sided α of 0.05 and 80% power, the calculated minimum sample size was 34 patients per group. This calculation was not based on the occurrence of postoperative SIRS. After application of the eligibility and exclusion criteria, the final analytical cohort comprised 150 patients, with 75 patients in each anaesthesia group.

### Perioperative assessment and data collection

Demographic characteristics, comorbidities, stone-related variables, laboratory parameters, anaesthetic technique, operative time, intraoperative fluid administration, irrigation volume, postoperative fever, SIRS, and length of hospital stay were prospectively recorded during the perioperative course and postoperative follow-up. Stone size was defined as the maximum stone diameter measured on preoperative imaging. The presence of staghorn calculi and residual stones was determined according to preoperative imaging findings and the intraoperative surgical assessment.

Midstream urine cultures were obtained from all patients within 7 days before surgery. The term “positive initial preoperative urine culture” refers to the initial screening culture obtained before antimicrobial treatment. Patients with a positive screening culture received culture-directed antimicrobial therapy based on susceptibility testing, after which a repeat urine culture was obtained. Elective PCNL was performed only after a sterile/negative repeat urine culture had been confirmed and there was no clinical evidence of active urinary tract infection. Perioperative antibiotic prophylaxis consisted of cefazolin 1 g administered intravenously approximately 15 min before surgical incision, in accordance with the institutional protocol. Postoperative urine and blood cultures were not collected as standardised study variables in all participants and therefore were not available for systematic cohort-level analysis.

Operative time was defined as the interval between the start and completion of the surgical procedure. The total volume of intraoperative crystalloid administered and the total irrigation volume used during the procedure were recorded. Irrigation was performed using a low-pressure gravity-driven system without active pressurisation. Direct intrarenal or renal pelvic pressure was not measured during the procedure.

### Surgical technique

All PCNL procedures were performed by the same experienced surgical team using a standardised technique. Following placement of a 6-Fr ureteral catheter by cystoscopy under either general or spinal anaesthesia, patients were positioned prone. Percutaneous renal access was established through an appropriate calyx under fluoroscopic guidance, and the tract was dilated using the standard technique. Stone fragmentation was performed with a pneumatic lithotripter and/or a holmium: YAG laser, followed by removal of stone fragments whenever feasible. Residual stone status was assessed by the operating surgeon at the end of the procedure, and a nephrostomy tube was inserted when indicated. In all patients, irrigation was provided using a low-pressure gravity-driven system without active pressurisation.

### Anaesthetic management

Spinal anaesthesia was performed at the L4–L5 intervertebral space under standard monitoring using 12.5 mg of hyperbaric bupivacaine. No additional sedation was administered in the spinal anaesthesia group. In the general anaesthesia group, anaesthesia was induced with propofol, fentanyl, and rocuronium and maintained with sevoflurane according to institutional practice. Standard perioperative haemodynamic monitoring was applied in all patients.

### Laboratory measurements and inflammatory indices

Blood samples were collected from all patients before surgery and at 6 h postoperatively. Haemoglobin, platelet, neutrophil, lymphocyte, and monocyte counts were obtained from complete blood count analyses, whereas serum albumin, lactate, and C-reactive protein (CRP) levels were determined by biochemical analysis.

The neutrophil-to-lymphocyte ratio (NLR) was calculated as neutrophils/lymphocytes; the systemic immune-inflammation index (SII) as platelets × neutrophils/lymphocytes; the systemic inflammation response index (SIRI) as neutrophils × monocytes/lymphocytes; and the aggregate index of systemic inflammation (AISI) as neutrophils × monocytes × platelets/lymphocytes. These indices were calculated using both the preoperative and postoperative 6-hour measurements. Delta (Δ) values were calculated by subtracting the preoperative value from the corresponding postoperative 6-hour value.

### Diagnostic criteria for SIRS and postoperative fever

Postoperative SIRS was defined as the presence of at least two of the following criteria: body temperature > 38 °C or < 36 °C; heart rate > 90 beats/min; respiratory rate > 20 breaths/min or PaCO₂ <32 mmHg; and white blood cell count > 12,000/µL or < 4,000/µL. Patients fulfilling SIRS criteria underwent clinical re-evaluation to assess whether the episode was attributable to the postoperative inflammatory process of interest or to a clearly identifiable alternative infectious or non-infectious cause. Adjudication was based on clinical examination and available laboratory findings, with additional imaging performed when clinically indicated. Imaging was not mandated uniformly for every SIRS episode but was guided by the clinical presentation. One patient whose postoperative SIRS was adjudicated as being attributable to an alternative cause was excluded from the final analytical cohort.

Patients were monitored for SIRS from the end of surgery through the first 24 postoperative hours. Vital signs, including body temperature, heart rate, and respiratory rate, were assessed routinely in the post-anaesthesia care unit and subsequently at least every 4 h on the surgical ward. Additional clinical assessments were performed whenever clinically indicated. A complete blood count, including the leukocyte count used as one of the SIRS criteria, was obtained at 6 h postoperatively.

Postoperative SIRS was ascertained on the basis of the predefined SIRS criteria documented during this 24-hour surveillance period. Postoperative fever, analysed as a separate secondary clinical outcome, was defined as a body temperature > 38 °C recorded during the first 24 postoperative hours. The exact onset time of individual SIRS episodes relative to the postoperative 6-hour blood sampling point was not recorded as a separate study variable.

### Outcomes

The primary outcome was the perioperative inflammatory response according to anaesthetic technique, operationalised as the between-group comparison of changes from preoperative values to postoperative 6-hour values of NLR, SII, SIRI, and AISI. Delta (Δ) values were calculated by subtracting the preoperative measurement from the corresponding postoperative 6-hour measurement.

Secondary clinical outcomes were postoperative SIRS, postoperative fever, and length of hospital stay. Exploratory secondary analyses evaluated the discriminatory performance of ΔNLR, ΔSII, ΔSIRI, and ΔAISI for postoperative SIRS and the associations between candidate perioperative variables and SIRS.

### Statistical analysis

Statistical analyses were performed using IBM SPSS Statistics for Windows, version 26.0 (IBM Corp., Armonk, NY, USA), and MedCalc version 22.026 (MedCalc Software Ltd., Ostend, Belgium). The distribution of continuous variables was assessed using the Shapiro–Wilk test. Non-normally distributed data are presented as median (interquartile range), and categorical variables as number (%). Between-group comparisons were performed using the Mann–Whitney U test, Pearson’s chi-square test, or Fisher’s exact test, as appropriate.

The primary outcome was the between-group comparison of perioperative changes in NLR, SII, SIRI, and AISI from the preoperative measurement to the postoperative 6-hour measurement. Postoperative SIRS was treated as a secondary clinical outcome, and ROC, decision curve, and regression analyses involving SIRS were considered exploratory secondary analyses. No separate event-based sample-size calculation was performed for postoperative SIRS; therefore, the study was not powered specifically for the development or validation of a SIRS prediction model. The diagnostic performance of inflammatory indices was evaluated using receiver operating characteristic (ROC) analysis. The area under the curve (AUC), optimal cut-off values, sensitivity, specificity, positive predictive value, and negative predictive value were calculated. Optimal cut-off values were derived from the present study cohort using the Youden index and were considered exploratory data-derived thresholds. Decision curve analysis (DCA) was performed to explore net benefit across a range of threshold probabilities.

Variables with *p* < 0.10 in univariable analyses were considered as candidates for exploratory multivariable analysis. Given the limited number of postoperative SIRS events, a deliberately parsimonious two-variable logistic regression model was constructed to reduce the risk of overfitting. Anaesthesia type was included as the principal clinical exposure of interest, and ΔAISI was selected as the inflammatory index with the highest discriminatory performance in ROC analysis. This multivariable analysis was considered exploratory and hypothesis-generating rather than a definitive prediction model. Given the limited number of SIRS events and the exploratory nature of the model, formal calibration analysis was not performed. Results are reported as adjusted odds ratios (ORs) with 95% confidence intervals (CIs). A two-sided *p* < 0.05 was considered statistically significant.

## Results

**Fig. 1:**
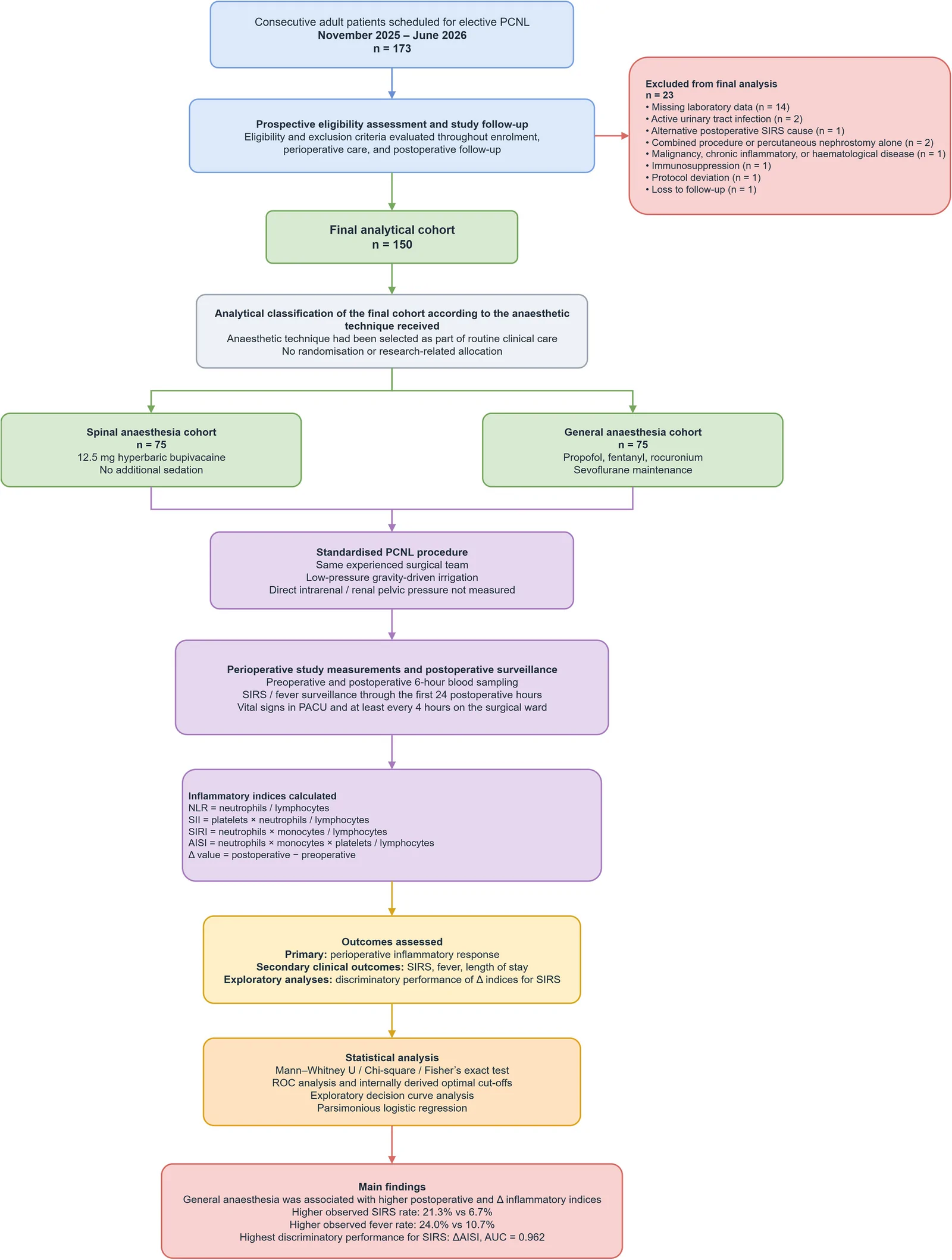
Study Flow Diagram. Flow diagram illustrating patient screening, eligibility assessment, exclusion process, and final classification according to the anaesthetic technique received

A total of 173 patients were assessed for eligibility. Of these, 23 were excluded: 14 because of missing laboratory data, 2 because of active urinary tract infection, 1 because a postoperative SIRS episode was clinically adjudicated as being attributable to an alternative cause, 2 because of a combined procedure or percutaneous nephrostomy alone, 1 because of malignancy, chronic inflammatory, or haematological disease, 1 because of immunosuppression, 1 because of a protocol deviation, and 1 because of loss to follow-up. The final analysis therefore included 150 patients: 75 in the spinal anaesthesia group and 75 in the general anaesthesia group (Fig. 1).

The demographic, clinical, and perioperative characteristics of the groups are shown in Table 1. There were no statistically significant differences between the spinal and general anaesthesia groups in age, body mass index, sex distribution, ASA physical status, diabetes mellitus, hypertension, hydronephrosis, operative time, preoperative albumin, lactate and CRP levels, irrigation volume, intraoperative fluid volume, positive initial preoperative urine culture, stone size, presence of staghorn calculi, or residual stone rates (all *p* > 0.05) (Table 1).

**Table 1:** Baseline and perioperative characteristics according to anaesthesia type

Variable	Spinal anaesthesia (*n* = 75)	General anaesthesia (*n* = 75)	*p* value
Age (years)	52.0 (46.5–58.5)	52.0 (45.0–58.5)	0.943
BMI (kg/m²)	27.2 (25.2–30.7)	26.5 (24.5–29.8)	0.320
Male sex, n (%)	35 (46.7%)	41 (54.7%)	0.414
ASA II, n (%)	54 (72.0%)	46 (61.3%)	0.225
ASA III, n (%)	21 (28.0%)	29 (38.7%)	0.225
Diabetes mellitus, n (%)	19 (25.3%)	15 (20.0%)	0.559
Hypertension, n (%)	25 (33.3%)	22 (29.3%)	0.725
Hydronephrosis, n (%)	23 (30.7%)	25 (33.3%)	0.861
Operation time (min)	84.0 (71.5–93.0)	88.0 (80.5–96.5)	0.107
Preoperative albumin (g/dL)	4.01 (3.81–4.23)	4.09 (3.92–4.33)	0.084
Preoperative lactate (mmol/L)	1.19 (1.06–1.40)	1.15 (0.96–1.33)	0.127
Preoperative CRP (mg/L)	8.30 (5.40–11.45)	7.50 (4.80–10.65)	0.159
Irrigation volume (mL)	8363.0 (5426.0 − 9291.0)	8313.0 (6258.0 − 10668.0)	0.379
Fluid volume (mL)	1932.0 (1900.0 − 2100.0)	1964.0 (1901.0 − 2077.0)	0.692
Positive initial preoperative urine culture, n (%)	16 (21.3%)	19 (25.3%)	0.629
Stone size (mm)	33.0 (26.0 − 38.0)	35.0 (24.0 − 40.0)	0.856
Staghorn stone: No	66 (88.0%)	61 (81.3%)	0.365
Staghorn stone: Yes	9 (12.0%)	14 (18.7%)	0.365
Residual stone, n (%)	20 (26.7%)	18 (24.0%)	0.851

Continuous variables are presented as median (interquartile range) and were compared using the Mann–Whitney U test. Categorical variables are presented as number (percentage) and were compared using the Pearson chi-square test or Fisher’s exact test, as appropriate

Positive initial preoperative urine culture refers to the screening culture obtained before culture-directed antimicrobial treatment. Patients with a positive screening culture underwent culture-directed antimicrobial therapy and repeat urine culture, and elective PCNL was performed only after a sterile/negative repeat culture had been confirmed

**Fig. 2:**
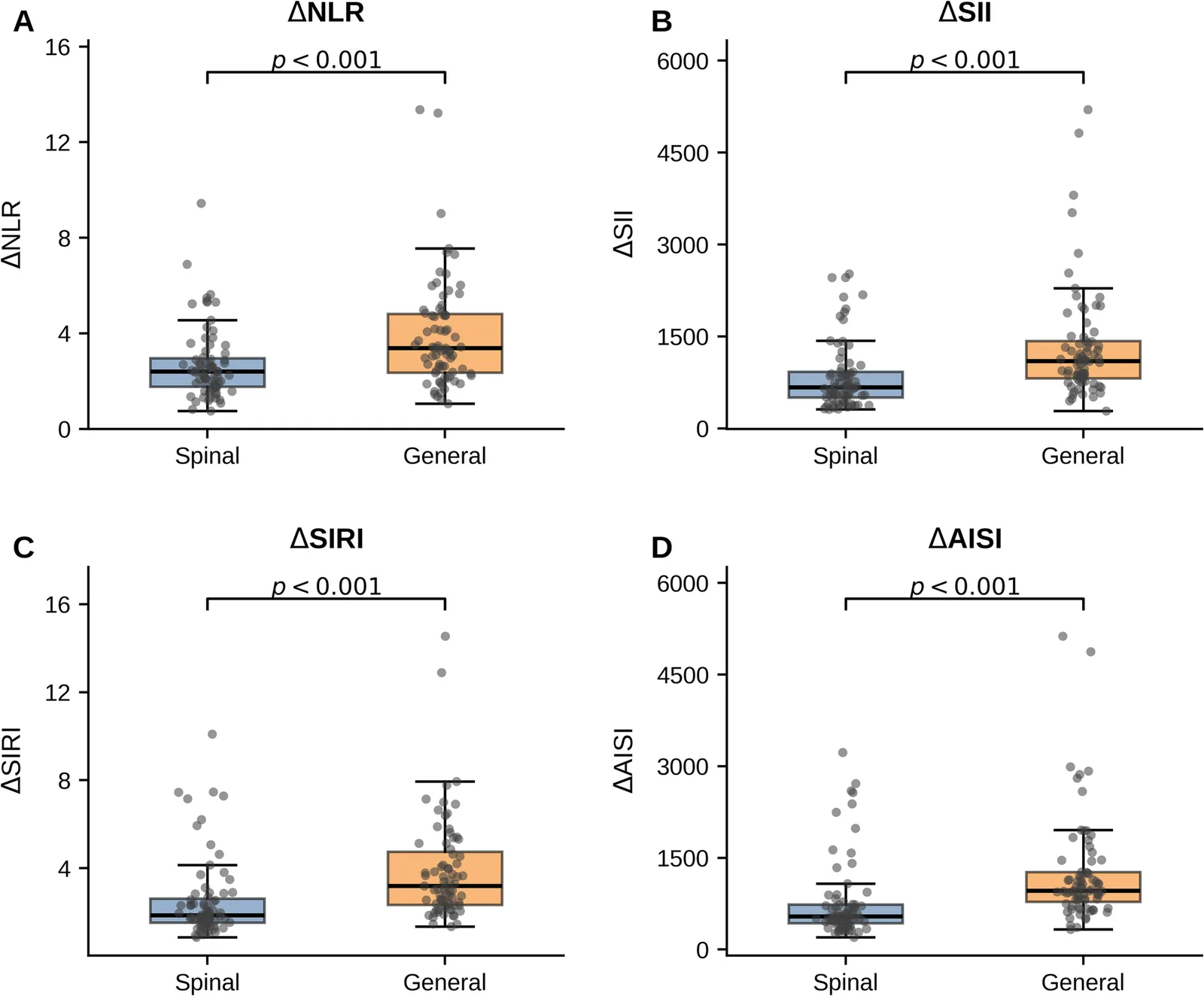
Postoperative changes in inflammatory indices in patients undergoing percutaneous nephrolithotomy according to anaesthesia type. Boxplots compare postoperative changes (Δ = postoperative − preoperative) in (**A**) neutrophil-to-lymphocyte ratio (ΔNLR), (**B**) systemic immune-inflammation index (ΔSII), (**C**) systemic inflammation response index (ΔSIRI), and (**D**) aggregate index of systemic inflammation (ΔAISI) between the spinal and general anaesthesia groups. Boxes indicate the interquartile range (IQR), the horizontal line within each box represents the median, whiskers extend to 1.5 × IQR, and individual circles represent individual patients. Between-group comparisons were performed using the Mann–Whitney U test

Preoperative inflammatory markers were similar between the two groups. In contrast, postoperative NLR, SII, SIRI, and AISI values were significantly higher in the general anaesthesia group than in the spinal anaesthesia group (all *p* < 0.001). Likewise, ΔNLR, ΔSII, ΔSIRI, and ΔAISI, which reflect perioperative changes, were significantly greater in the general anaesthesia group (all *p* < 0.001) (Table 2; Fig. 2).

**Table 2:** Comparison of preoperative and postoperative inflammatory indices between anaesthesia groups

Inflammatory marker	Spinal anaesthesia (*n* = 75)	General anaesthesia (*n* = 75)	*p* value
NLR preoperative	2.33 (2.03–2.62)	2.36 (2.07–2.73)	0.582
NLR postoperative	4.66 (3.88–5.66)	5.92 (4.71–7.45)	< 0.001
ΔNLR	2.40 (1.76–2.96)	3.39 (2.36–4.81)	< 0.001
SII preoperative	578.74 (476.47–624.39)	573.98 (480.11–637.70)	0.955
SII postoperative	1226.01 (980.16–1594.69)	1698.08 (1324.53–2050.94)	< 0.001
ΔSII	669.61 (505.98–923.32)	1100.53 (819.57–1424.65)	< 0.001
SIRI preoperative	1.10 (0.87–1.34)	1.21 (0.99–1.40)	0.197
SIRI postoperative	3.03 (2.51–4.05)	4.63 (3.56–6.04)	< 0.001
ΔSIRI	1.86 (1.53–2.62)	3.19 (2.34–4.74)	< 0.001
AISI preoperative	259.20 (199.15–354.48)	287.59 (229.13–356.36)	0.489
AISI postoperative	842.48 (635.19–1124.44)	1318.62 (1043.77–1600.24)	< 0.001
ΔAISI	542.07 (430.40–735.86)	963.10 (780.49–1265.76)	< 0.001

Continuous variables are presented as median (IQR). Δ values represent postoperative minus preoperative changes. Between-group comparisons were performed using the Mann–Whitney U test. NLR, neutrophil-to-lymphocyte ratio; SII, systemic immune-inflammation index; SIRI, systemic inflammation response index; AISI, aggregate index of systemic inflammation

Postoperative clinical outcomes are summarised in Table 3. Postoperative SIRS occurred in 5 patients (6.7%) in the spinal anaesthesia group and in 16 patients (21.3%) in the general anaesthesia group; this difference was statistically significant (*p* = 0.017). Postoperative fever was also more frequent in the general anaesthesia group than in the spinal anaesthesia group (24.0% vs. 10.7%, *p* = 0.031). Length of hospital stay did not differ significantly between the groups (*p* = 0.615) (Table 3).

**Table 3:** Postoperative Clinical Outcomes and Complications According to Anaesthesia Type

Clinical outcome	Spinal anaesthesia (*n* = 75)	General anaesthesia (*n* = 75)	*p* value
Postoperative SIRS: No	70 (93.3%)	59 (78.7%)	
Postoperative SIRS: Yes	5 (6.7%)	16 (21.3%)	0.017
Postoperative fever: No	67 (89.3%)	57 (76.0%)	
Postoperative fever: Yes	8 (10.7%)	18 (24.0%)	0.031
Length of hospital stay (days)	4.0 (3.0–5.0)	4.0 (3.0–5.0)	0.615

Continuous variables are presented as median (interquartile range [IQR]), and categorical variables as number (%). Continuous variables were compared using the Mann–Whitney U test. Categorical variables were compared using the Pearson chi-square test or Fisher’s exact test, as appropriate

Postoperative fever was defined as a body temperature > 38 °C recorded during the first 24 postoperative hours

**Fig. 3:**
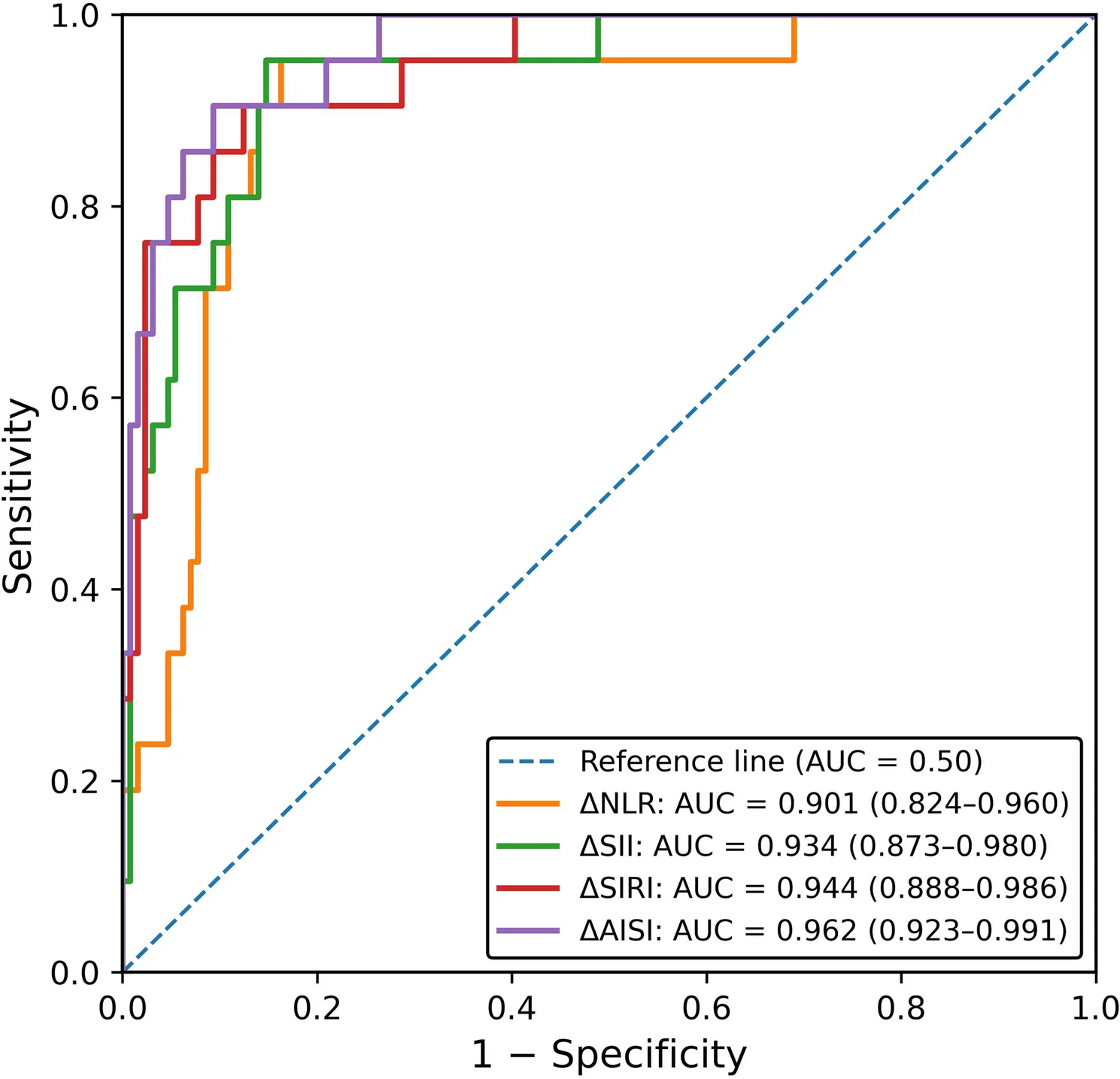
Receiver Operating Characteristic (ROC) Curves for Discrimination of Postoperative SIRS. Receiver operating characteristic curves demonstrating the discriminatory performance of ΔNLR, ΔSII, ΔSIRI, and ΔAISI for postoperative SIRS. Optimal cut-off values were determined using the Youden index. AUC values with 95% confidence intervals are presented in Table 4

In ROC analysis evaluating discrimination of postoperative SIRS, all evaluated inflammatory indices showed high discriminatory performance (Table 4; Fig. 3). The highest performance was observed for ΔAISI (AUC = 0.962, 95% CI: 0.923–0.991), followed by ΔSIRI (AUC = 0.944), ΔSII (AUC = 0.934), and ΔNLR (AUC = 0.901). The internally derived optimal cut-off value for ΔAISI was 1264.60, with an apparent sensitivity of 90.5% and specificity of 90.7% in the present cohort. In an exploratory analysis, the combined model incorporating ΔAISI and anaesthesia type yielded an AUC of 0.967 in the present cohort, compared with 0.962 for ΔAISI alone. Because both estimates were obtained from the same dataset and no formal statistical comparison between the AUCs was performed, this small numerical difference should not be interpreted as evidence of superior discriminatory performance.

**Table 4:** Receiver Operating Characteristic (ROC) Analysis for Discrimination of Postoperative SIRS

Variable	AUC	95% CI	Optimal cut-off	Sensitivity	Specificity
ΔNLR	0.901	0.824–0.960	3.84	95.2%	83.7%
ΔSII	0.934	0.873–0.980	1280.48	95.2%	85.3%
ΔSIRI	0.944	0.888–0.986	3.99	90.5%	87.6%
ΔAISI	0.962	0.923–0.991	1264.60	90.5%	90.7%

Receiver operating characteristic (ROC) analyses were performed to evaluate the discriminatory performance of postoperative inflammatory markers for postoperative SIRS. Optimal cut-off values were derived from the present cohort using the Youden index and should be regarded as exploratory data-derived thresholds. AUC values are presented with 95% confidence intervals. Δ values represent postoperative minus preoperative changes

**Fig. 4:**
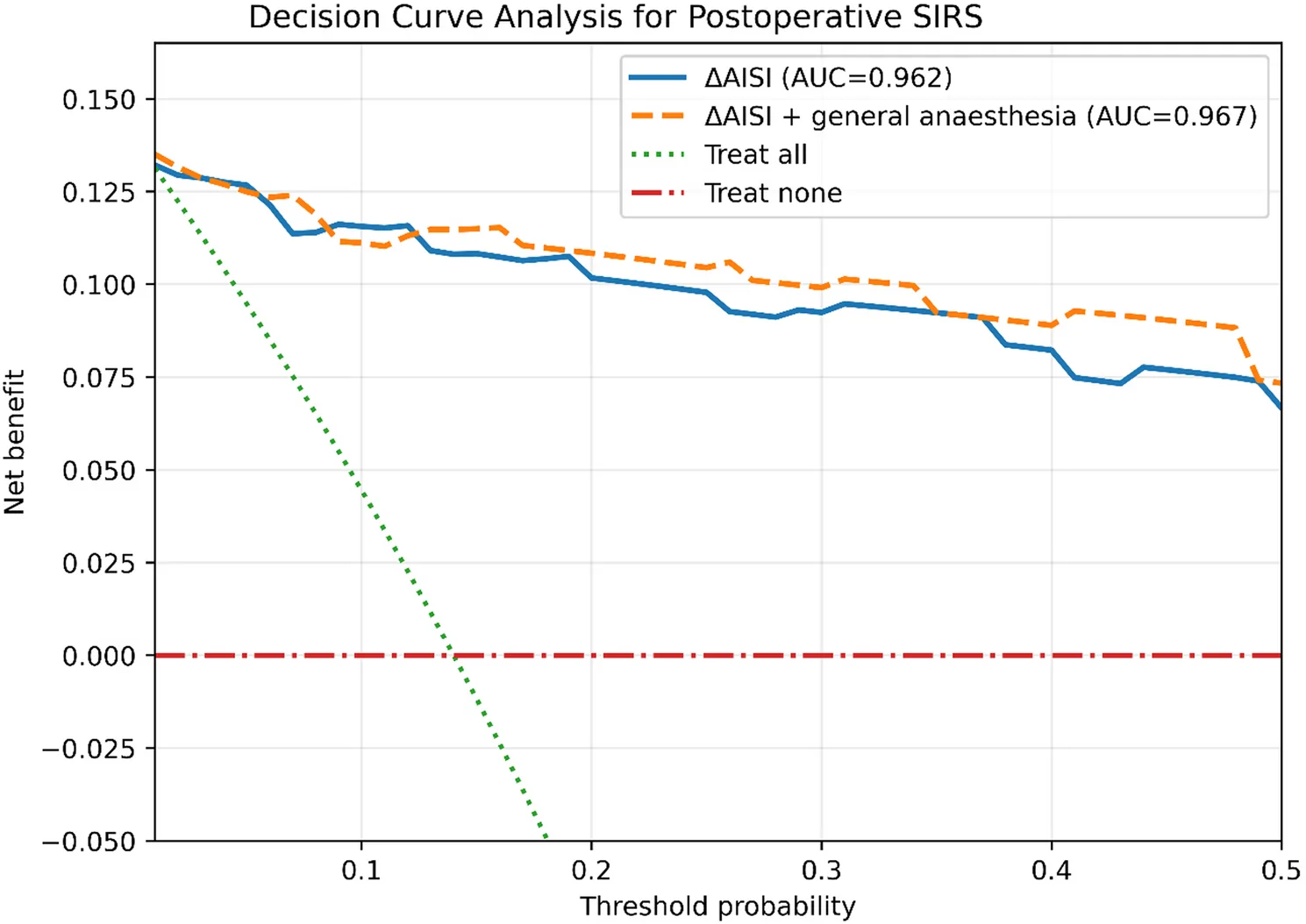
Decision Curve Analysis of ΔAISI Alone and the Combined ΔAISI Plus Anaesthesia Model for Postoperative SIRS. Decision curve analysis comparing the observed net benefit of ΔAISI alone with a combined model including ΔAISI and anaesthesia type in relation to postoperative SIRS. The combined model showed a modest increase in observed net benefit over ΔAISI alone at some intermediate threshold probabilities in the present cohort

In exploratory decision curve analysis, ΔAISI showed the highest observed net benefit among the evaluated inflammatory indices across a range of threshold probabilities in the present cohort. The model incorporating ΔAISI and anaesthesia type showed a modest increase in net benefit compared with ΔAISI alone at some intermediate threshold probabilities (Fig. 4; Supplementary Figure S1). These findings should be interpreted as exploratory because the analyses were performed and evaluated within the same cohort.

In univariable logistic regression analysis, general anaesthesia, operative time, ΔNLR, ΔSII, and ΔAISI were significantly associated with postoperative SIRS (Supplementary Table 1). Diagnostic performance metrics are presented in Supplementary Table 2. Given that only 21 postoperative SIRS events occurred, an exploratory parsimonious multivariable logistic regression model including only anaesthesia type and ΔAISI was fitted to limit the risk of overfitting. In this exploratory model, general anaesthesia (adjusted OR = 12.02, 95% CI: 1.06–136.68, *p* = 0.045) and ΔAISI (adjusted OR = 1.51 per 100-unit increase, 95% CI: 1.26–1.81, *p* < 0.001) were associated with postoperative SIRS after mutual adjustment (Supplementary Table 3). The very wide confidence interval for general anaesthesia indicates substantial imprecision in the magnitude of this association and warrants cautious interpretation.

## Discussion

### Main findings

This prospective observational study compared the early postoperative inflammatory response between patients undergoing elective percutaneous nephrolithotomy under general versus spinal anaesthesia. The main finding was that, although preoperative inflammatory markers were similar between the groups, postoperative NLR, SII, SIRI, and AISI values, as well as the Δ values reflecting perioperative changes in these indices, were significantly higher in the general anaesthesia group. Postoperative SIRS and fever were also more frequent among patients who received general anaesthesia. Length of hospital stay, however, did not differ between the groups.

Another relevant finding was that ΔAISI showed the highest discriminatory performance for postoperative SIRS among the inflammatory indices evaluated. In ROC analysis, ΔAISI had greater discriminatory ability than the other inflammatory markers, and in the exploratory parsimonious multivariable logistic regression model, both general anaesthesia and increasing ΔAISI were associated with postoperative SIRS after mutual adjustment. These findings demonstrate an association between anaesthetic technique and the magnitude of the early postoperative inflammatory response, although the biological mechanisms underlying this association cannot be determined from the present study.

Although no statistically significant differences were observed between the groups in several measured clinical, stone-related, and perioperative variables, residual differences in measured or unmeasured confounders cannot be excluded because of the non-randomised design. The more pronounced inflammatory response observed in the general anaesthesia group should therefore be interpreted as an association that warrants further investigation rather than as evidence of an effect caused by anaesthetic technique.

### Comparison with previous studies

The inflammatory response after PCNL is multifactorial and differs in important respects from that observed in many other surgical settings. In addition to general surgical tissue injury, PCNL involves procedure-specific factors such as renal parenchymal access, tract dilation, irrigation-related factors, and potential bacterial or endotoxin translocation, each of which may contribute to postoperative systemic inflammation [10–12]. In the present study, irrigation was gravity-driven without active pressurisation, and direct intrarenal or renal pelvic pressure was not measured; therefore, the contribution of pressure-related mechanisms cannot be quantified from our data. These PCNL-specific factors should be considered when interpreting the observed inflammatory differences between anaesthesia groups and limit attribution of those differences to anaesthetic technique alone.

In the present study, preoperative inflammatory indices were comparable between the two groups, whereas postoperative NLR, SII, SIRI, and AISI values and their perioperative changes were higher in the general anaesthesia group. The higher rates of SIRS and fever in patients receiving general anaesthesia further support an association between general anaesthesia and a more marked systemic inflammatory response after PCNL. The similarity between groups in stone size, staghorn calculi, operative time, irrigation volume, and positive urine culture suggests that this difference cannot be explained solely by surgical complexity.

Previous studies have reported associations between anaesthetic technique and the perioperative inflammatory response in other surgical populations [13, 14]. In total knee arthroplasty, spinal anaesthesia has been associated with lower postoperative NLR, SII, and other systemic inflammatory markers compared with general anaesthesia [15, 16]. These observations provide relevant contextual support for the possibility that anaesthetic technique may be associated with differences in postoperative systemic inflammation. However, direct extrapolation from orthopaedic surgery to PCNL should be made cautiously because the two procedures represent distinct surgical and inflammatory settings. In addition to tissue injury and perioperative stress responses common to surgery, PCNL involves procedure-specific factors such as renal parenchymal access, tract dilation, irrigation-related factors, and potential bacterial or endotoxin translocation. Accordingly, the present findings extend investigation of the association between anaesthetic technique and inflammatory indices to PCNL but do not establish that the mechanisms reported in orthopaedic populations are directly transferable to urological surgery.

Most previous PCNL studies have focused on risk factors for postoperative sepsis or SIRS, without examining inflammatory indices in relation to anaesthetic technique [17–19]. By evaluating complete blood count–derived inflammatory markers, including NLR, SII, SIRI, and especially AISI, in the context of spinal versus general anaesthesia, the present study adds a clinically relevant perspective to the existing literature.

### Clinical relevance of ΔAISI in relation to postoperative SIRS

Among the inflammatory indices evaluated in this study, ΔAISI showed the highest discriminatory performance for postoperative SIRS. Because AISI incorporates neutrophils, monocytes, platelets, and lymphocytes into a single formula, it may reflect several cellular components of the inflammatory response at the same time [5, 6]. ΔAISI may therefore provide a broader measure of systemic inflammation than NLR, which primarily reflects the balance between neutrophils and lymphocytes.

The high AUC observed for ΔAISI in ROC analysis indicates strong discriminatory capacity for early postoperative SIRS after PCNL. However, diagnostic performance should not be interpreted on the basis of AUC alone. In the present cohort, ΔAISI values below the internally derived cut-off were associated with a high negative predictive value; however, because the threshold was selected from the same dataset in which its performance was evaluated, this finding should be considered exploratory. The threshold should therefore not be regarded as an established clinical decision cut-off until its performance has been confirmed in an independent cohort. By contrast, the more limited positive predictive value indicates that an elevated ΔAISI alone is not sufficient to establish a diagnosis of SIRS and should be interpreted together with clinical findings, vital signs, and other laboratory results.

In the exploratory parsimonious multivariable logistic regression model, higher ΔAISI remained associated with postoperative SIRS after adjustment for anaesthesia type. This association supports further evaluation of ΔAISI as a potential adjunctive inflammatory marker. However, because the exact temporal relationship between the postoperative 6-hour measurement and SIRS onset was not established in all patients and no independent validation was performed, the present findings do not establish prospective predictive validity or clinical utility. The very wide confidence interval for general anaesthesia (adjusted OR = 12.02, 95% CI: 1.06–136.68) indicates substantial imprecision in the estimated magnitude of the association, which is likely related to the limited number of postoperative SIRS events. Accordingly, the point estimate should not be interpreted as providing a precise measure of effect size, and the regression findings should be considered exploratory and hypothesis-generating pending confirmation in larger cohorts.

In exploratory decision curve analysis, ΔAISI showed the highest observed net benefit among the evaluated inflammatory indices across a range of threshold probabilities in the present cohort. Adding anaesthesia type to ΔAISI produced a modest increase in net benefit at some intermediate threshold probabilities. However, because these analyses were performed and evaluated within the same cohort, they should not be interpreted as establishing clinical utility and require confirmation in an independent population.

### Potential biological context

Surgical trauma is accompanied by activation of inflammatory and neuroendocrine pathways, including changes in innate immune activity and circulating leukocyte populations [7, 8]. Previous studies have suggested that anaesthetic technique may be associated with differences in the magnitude of this perioperative stress response. These mechanisms provide biological context for the observed findings but should not be interpreted as pathways demonstrated by the present study.

Previous literature has reported differences in neuroendocrine and inflammatory responses between regional and general anaesthesia, including potential differences in sympathetic activation and perioperative immune responses [7, 8, 20, 21]. Such mechanisms could theoretically contribute to differences in postoperative haematological inflammatory indices. However, the present observational findings do not establish that these pathways mediated the differences observed between the anaesthesia groups.

PCNL itself may generate an inflammatory response through several procedure-specific mechanisms, including renal parenchymal injury, tract dilation, irrigation-related factors, and potential bacterial or endotoxin translocation [9–12, 19]. Although several measured stone-related and perioperative variables were similar between the groups in the present cohort, these observations do not exclude differences in other measured or unmeasured factors. Consequently, the mechanisms underlying the observed between-group differences in inflammatory indices cannot be determined from the present observational data.

### Clinical implications

The findings of this study suggest that anaesthetic technique may be associated with differences in the early postoperative inflammatory response in patients undergoing PCNL. The higher inflammatory indices and higher rates of SIRS and fever in the general anaesthesia group indicate that anaesthetic technique may be one of the factors considered during perioperative assessment, particularly in patients at increased risk of inflammatory complications. However, anaesthetic technique should not be used as the sole determinant of clinical decision-making and should be considered together with patient characteristics, surgical requirements, and contraindications.

Because ΔAISI can be calculated from a routine complete blood count without additional testing, its observed discriminatory performance warrants further investigation as a potential adjunctive marker. However, its clinical role remains uncertain and requires independent validation before use for postoperative risk stratification. Elevated ΔAISI values, however, should not be used in isolation and must be interpreted alongside clinical findings and other laboratory results.

Although postoperative SIRS and fever were more frequent in the general anaesthesia group, length of hospital stay was similar between the groups. These findings are not necessarily contradictory because SIRS and fever represent early postoperative inflammatory events, whereas hospital stay is a broader downstream outcome influenced by multiple factors, including haemodynamic stability, pain control, mobilisation, surgical recovery, postoperative complications, and institutional discharge criteria. The present study was not designed to determine whether individual SIRS episodes directly delayed discharge, and no analysis linking the severity or duration of individual inflammatory episodes to length of stay was performed. Therefore, the absence of a between-group difference in hospital stay should not be interpreted as evidence that the observed inflammatory differences lacked clinical relevance.

### Strengths

The main strengths of this study are its prospective design, single-centre setting, and assessment of PCNL patients undergoing spinal or general anaesthesia under standardised surgical and anaesthetic protocols.

All procedures were performed by the same experienced surgical team, and no statistically significant between-group differences were observed in several measured baseline and perioperative characteristics. These features reduced procedural heterogeneity, although residual and unmeasured confounding cannot be excluded.

In addition, the inflammatory response was assessed not through a single biomarker but through complementary complete blood count–derived indices, including NLR, SII, SIRI, and AISI. The use of preoperative and postoperative 6-hour measurements allowed objective evaluation of perioperative changes. ROC analysis, exploratory decision curve analysis, and a parsimonious multivariable model were used to characterise discriminatory performance, observed net benefit, and adjusted associations within the present cohort.

### Limitations

This study has several limitations. First, it was conducted at a single centre and employed a prospective, non-randomised observational design. Anaesthetic technique was selected as part of routine clinical care rather than through randomisation. Although no statistically significant between-group differences were observed in several measured baseline clinical, stone-related, and perioperative variables, this does not establish equivalence between the groups with respect to all potential confounders. Residual and unmeasured confounding, as well as treatment-selection bias inherent to the non-randomised design, cannot be excluded. Factors that are difficult to quantify, including clinical judgement, anaesthesiologist preference, surgical assessment, and patient preference, may also have influenced the choice of anaesthesia. Propensity-score adjustment was not prespecified and was not introduced post hoc during revision. Although such methods may reduce confounding from measured covariates, they cannot account for unmeasured factors involved in clinical selection of anaesthetic technique. Therefore, the findings should be interpreted as associations rather than evidence of a causal effect of anaesthetic technique.

Second, one patient was excluded from the final analytical cohort after a postoperative SIRS episode was clinically adjudicated as being attributable to an alternative cause. Exclusion after occurrence of the outcome may introduce outcome-based selection and may reduce generalisability. In addition, although patients fulfilling SIRS criteria underwent clinical assessment for alternative infectious and non-infectious causes, it cannot be confirmed with certainty that all SIRS episodes retained in the analytical cohort were attributable solely to the postoperative inflammatory process of interest.

Third, although postoperative SIRS was systematically assessed during the first 24 postoperative hours, the exact timing of individual SIRS episodes relative to the postoperative 6-hour blood sample was not recorded as a separate study variable. Consequently, temporal precedence of the 6-hour inflammatory indices over SIRS cannot be established in all patients, and the SIRS-related biomarker analyses should be interpreted in terms of discrimination and association rather than prospective predictive validity.

Fourth, only 21 postoperative SIRS events occurred in the final cohort, and the study was not powered specifically for SIRS-related modelling. The limited number of events restricts statistical precision and model stability. Accordingly, the ROC, decision curve, and regression analyses involving SIRS should be regarded as exploratory and hypothesis-generating rather than as development or validation of a definitive prediction model. A deliberately parsimonious exploratory logistic regression model containing only two variables was used to limit the risk of overfitting. Formal model calibration was not assessed. In addition, the ROC cut-off values were derived and evaluated within the same cohort, which may result in optimistic estimates of classification performance, and no independent validation cohort was available. The very wide 95% confidence interval for general anaesthesia (1.06–136.68) further demonstrates substantial imprecision and precludes precise estimation of the magnitude of this association.

Fifth, the inflammatory response was assessed only preoperatively and at 6 h postoperatively. The temporal kinetics and peak values of the inflammatory indices could therefore not be determined. Cortisol, catecholamines, inflammatory cytokines, and other direct markers of neuroendocrine or immune activation were not measured; consequently, the biological pathways potentially underlying the observed between-group differences cannot be established from the present data. Serial biomarker measurements at additional postoperative time points would provide a more complete assessment of the inflammatory response.

Finally, the study focused on early postoperative inflammatory changes and short-term clinical outcomes. Longer-term outcomes, including subsequent infectious complications, readmission, renal function, and mortality, were not systematically evaluated. Larger multicentre studies with adequate numbers of SIRS events, prespecified adjustment strategies, serial biomarker assessment, and independent validation are required to confirm these findings.

### Future perspectives

The findings of this study warrant further investigation of the association between anaesthetic technique and the early postoperative inflammatory response in patients undergoing PCNL through larger, multicentre, and preferably randomised controlled studies. Serial assessment of inflammatory biomarkers at multiple postoperative time points, together with established biomarkers such as procalcitonin, interleukins, and other cytokines, may provide a more comprehensive understanding of the temporal and biological characteristics of the postoperative inflammatory response [3, 4]. Independent external validation of ΔAISI and the internally derived cut-off is required in larger, preferably multicentre cohorts with an adequate number of SIRS events. Such studies should assess discrimination, calibration, and clinical utility before any threshold derived from the present cohort is considered for clinical implementation.

## Conclusion

In conclusion, general anaesthesia was associated with a more pronounced early postoperative systemic inflammatory response and higher observed rates of SIRS and fever than spinal anaesthesia in patients undergoing elective PCNL. These findings should be interpreted as associations rather than evidence of a causal effect of anaesthetic technique.

Among the inflammatory indices evaluated, ΔAISI showed the highest discriminatory performance for postoperative SIRS within the present cohort and remained associated with SIRS after adjustment for anaesthesia type in the exploratory parsimonious model. However, the SIRS-related analyses were based on only 21 events, the temporal relationship between the postoperative 6-hour biomarker measurement and SIRS onset was not established in all patients, and the reported threshold was internally derived without independent validation.

Accordingly, these findings should be considered exploratory and hypothesis-generating. Larger independent multicentre studies with adequate numbers of outcome events, serial biomarker assessment, and formal validation are required before ΔAISI or its derived threshold can be considered for clinical risk stratification after PCNL.

## Supplementary Information

Supplementary Material 1.

## Data Availability

The datasets generated and/or analyzed during the current study are not publicly available due to institutional and ethical restrictions but are available from the corresponding author on reasonable request.
