# Supplementary material for: Early systemic inflammatory indices and postoperative SIRS after percutaneous nephrolithotomy under spinal versus general anaesthesia: a prospective cohort study
Source: BMC Urol. 2026 Sep 19;26:228. doi: 10.1186/s12894-026-02368-y (PMC13613588; doi:10.1186/s12894-026-02368-y)
Supplement: Supplementary file 1 [file 12894_2026_2368_MOESM1_ESM.pdf]

Supplementary

Supplementary Figure S1. Decision Curve Analysis of Postoperative Inflammatory Indices for Postoperative SIRS

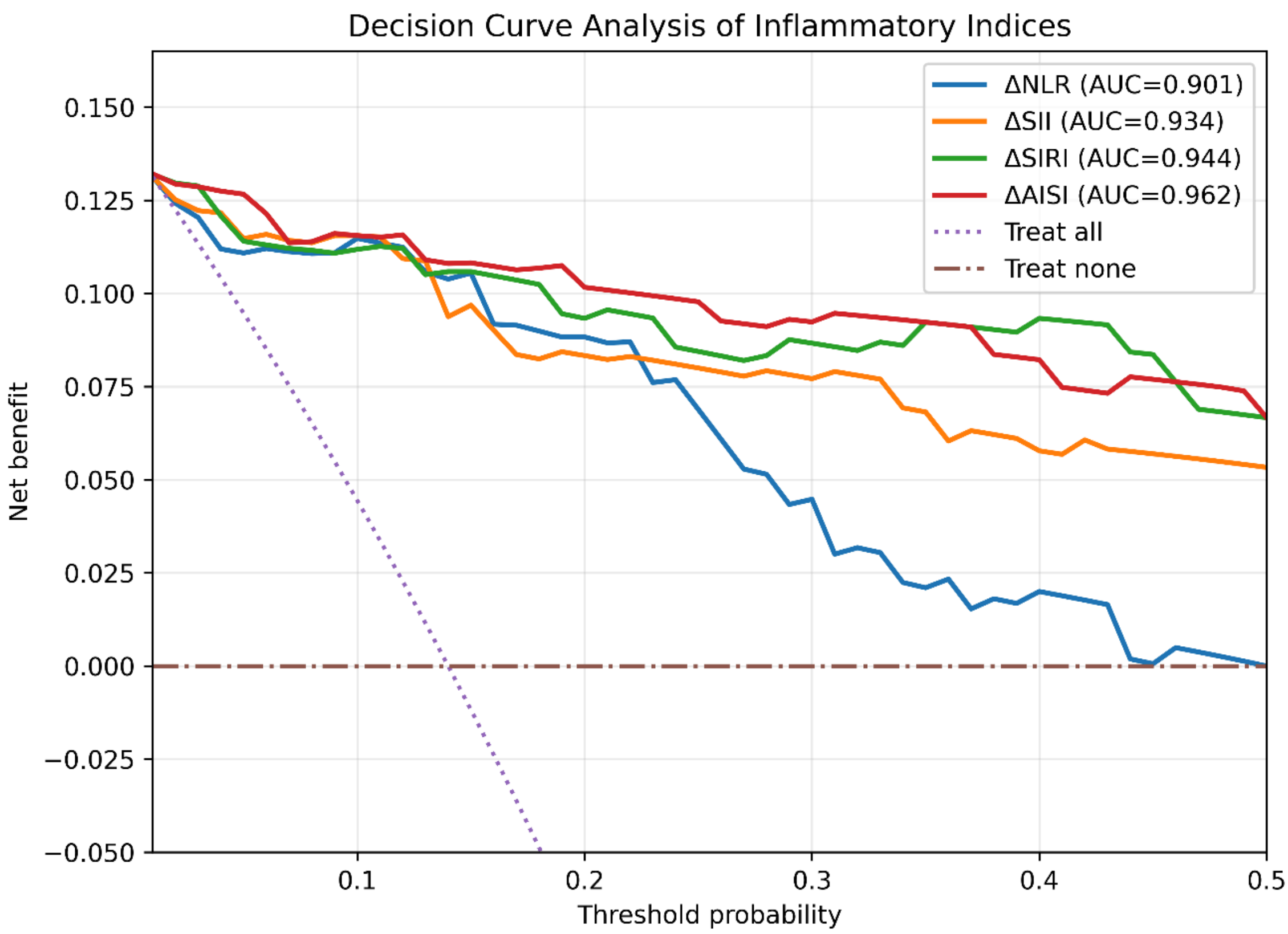

Decision curve analysis comparing the observed net benefit of postoperative changes in inflammatory indices (ΔNLR, ΔSII, ΔSIRI, and ΔAISI) in relation to postoperative systemic inflammatory response syndrome (SIRS) after percutaneous nephrolithotomy. Across the evaluated threshold probabilities in the present cohort, ΔAISI showed the highest observed net benefit, followed by ΔSIRI and ΔSII, whereas ΔNLR showed lower observed net benefit. The "Treat all" and "Treat none" strategies are shown as reference curves.

Supplementary Table 1. Univariable Logistic Regression Analysis of Factors Associated with Postoperative SIRS

Variable	OR	95% CI	p value
General anaesthesia (vs spinal)	3.80	1.31–10.98	0.014
Age (per year)	1.01	0.97–1.06	0.578
BMI (per kg/m²)	1.09	0.96–1.23	0.182
Diabetes mellitus	0.15	0.02–1.13	0.065
Operation time (per 10 min)	1.60	1.19–2.14	0.002
Preoperative albumin (per 1 g/dL)	0.91	0.23–3.65	0.895
ΔNLR (per unit)	2.41	1.69–3.45	<0.001
ΔSII (per 100 units)	1.31	1.18–1.45	<0.001
ΔAISI (per 100 units)	1.41	1.24–1.61	<0.001

Supplementary Table 2. Discriminatory performance of inflammatory markers for postoperative SIRS

Marker	Optimal cut-off	Sensitivity	Specificity	PPV	NPV	LR+	LR−
ΔNLR	3.84	95.2%	83.7%	48.8%	99.1%	5.85	0.06
ΔSII	1280.48	95.2%	85.3%	51.3%	99.1%	6.47	0.06
ΔSIRI	3.99	90.5%	87.6%	54.3%	98.3%	7.29	0.11
ΔAISI	1264.60	90.5%	90.7%	61.3%	98.3%	9.73	0.11

Supplementary Table 3. Exploratory Parsimonious Multivariable Logistic Regression Analysis of Factors Associated with Postoperative SIRS

Variable	Adjusted OR	95% CI	p value
General anaesthesia (vs spinal)	12.02	1.06–136.68	0.045
ΔAISI, per 100-unit increase	1.51	1.26–1.81	<0.001

A parsimonious multivariable logistic regression model was constructed to minimize the risk of overfitting due to the limited number of postoperative SIRS events. Only anaesthesia type and ΔAISI were included in the final model. ΔAISI was entered per 100-unit increase. OR, odds ratio; CI, confidence interval; AISI, aggregate index of systemic inflammation; SIRS, systemic inflammatory response syndrome.
